# Does ADHD worsen inhibitory control in preschool children born very premature and/or with very low birth weight?

**DOI:** 10.1590/2237-6089-2019-0075

**Published:** 2020-11-17

**Authors:** Bárbara Calil Lacerda, Sophia B.S. Martínez, Adelar Pedro Franz, Carlos Renato Moreira-Maia, Rita C. Silveira, Renato S. Procianoy, Luis A. Rohde, Flávia Wagner

**Affiliations:** 1 Programa de Pós-Graduação em Psiquiatria Universidade Federal do Rio Grande do Sul Porto AlegreRS Brazil Programa de Pós-Graduação em Psiquiatria, Universidade Federal do Rio Grande do Sul (UFRGS), Porto Alegre, RS, Brazil.; 2 Programa de Transtorno de Déficit de Atenção/Hiperatividade Hospital de Clínicas de Porto Alegre Porto AlegreRS Brazil Programa de Transtorno de Déficit de Atenção/Hiperatividade (ProDAH), Hospital de Clínicas de Porto Alegre, Porto Alegre, RS, Brazil.; 3 Departamento de Pediatria UFRGS Porto AlegreRS Brazil Departamento de Pediatria, UFRGS, Porto Alegre, RS, Brazil.; 4 Serviço de Neonatologia Hospital de Clínicas de Porto Alegre Porto AlegreRS Brazil Serviço de Neonatologia, Hospital de Clínicas de Porto Alegre, Porto Alegre, RS, Brazil.; 5 Serviço de Psiquiatria da Infância e Adolescência Hospital de Clínicas de Porto Alegre UFRGS Porto AlegreRS Brazil Departamento de Psiquiatria, Serviço de Psiquiatria da Infância e Adolescência, Hospital de Clínicas de Porto Alegre, UFRGS, Porto Alegre, RS, Brazil.; 6 Instituto Nacional de Psiquiatria do Desenvolvimento para a Infância e Adolescência UFRGS Porto AlegreRS Brazil Instituto Nacional de Psiquiatria do Desenvolvimento para a Infância e Adolescência, UFRGS, Porto Alegre, RS, Brazil.

**Keywords:** Inhibitory control, prematurity, preschool, ADHD

## Abstract

**Introduction:**

Deficits in executive functioning, especially in inhibitory control, are present in children born very premature and/or with very low birth weight (VP/VLBW) and in children with attention-deficit/hyperactivity disorder (ADHD).

**Objective:**

To evaluate whether ADHD imposes additional inhibitory control (IC) deficits in preschoolers born VP/VLBW.

**Methods:**

79 VP/VLBW (4 to 7 years) children were assessed for ADHD using the Schedule for Affective Disorders and Schizophrenia for School Aged Children – Present and Lifetime Version (K-SADS-PL). IC was measured with Conners’ Kiddie Continuous Performance Test (K-CPT 2) and the Behavior Rating Inventory of Executive Function – Preschool Version (BRIEF-P).Results: No significant differences were found between ADHD (n = 24) and non-ADHD children (n = 55) for any of the measures (p = 0.062 to p = 0.903). Both groups had deficits in most K-CPT 2 scores compared to normative samples, indicating poor IC and inconsistent reaction times.

**Conclusions:**

ADHD does not aggravate IC deficits in VP/VLBW children. Either neuropsychological tasks and parent reports of executive functions (EFs) may not be sensitive enough to differentiate VP/VLBW preschoolers with and without ADHD, or these children’s EFs are already so impaired that there is not much room for additional impairments imposed by ADHD.

## Introduction

Preterm birth is defined as birth occurring before 37 full weeks of gestation. Around the world, its prevalence ranges from 5% to 18%.^1^ There are sub-categories of preterm birth, based on gestational age: extremely premature (EP): < 28 weeks; very premature (VP): 28 to < 32 weeks, and moderate to late preterm infants (MLP): 32 to < 37 weeks of gestation. Prematurity can also be classified according to birth weight: extremely low birth weight (ELBW): < 1,000 grams and very low birth weight (VLBW): 1,000 to < 1,500 grams.^1 , 2^ Our study will focus on very preterm and/or very low birth weight (VP/VLBW) children.

Although the survival rates for VP and VLBW babies have increased over recent years due to advances in perinatal and neonatal care,^3^ these groups are still at high risk of death and disability.^2^ For instance, several structures of the central nervous system may be compromised in VP/VLBW children.^4^ Abnormalities of brain structure may, in turn, translate to neuropsychological deficits in executive functions.^3^

Prematurity is frequently associated with neurodevelopmental disorders, such as ADHD.^5^ This disorder is characterized by inattention and/or hyperactivity/impulsivity interfering with functioning.^6^ The validity of ADHD diagnosis in preschool children has been established.^7^ Overall, neuropsychological studies of preschoolers with ADHD show similar results to studies of school-aged children,^8^ including deficits in response inhibition, delay aversion, working memory, and sustained attention.^9 , 10^ Inhibitory control is an executive control function that enables an ongoing response to be stopped. It enables inhibition and alteration of inappropriate strategies, such as detecting and correcting an error in an academic task.^11^ Preschool children with ADHD tend to perform significantly worse in inhibitory control tasks and inventories, when compared to those without the disorder.^12 - 14^

Although ADHD is a prevalent disorder in children born prematurely and/or at very low birth weight,^3 , 15^ few studies have investigated whether ADHD is associated with additional impairment of inhibitory control performances in premature children. To our knowledge, only one study has compared preterm children with and without ADHD using a neuropsychological task. Both neurophysiological and neuropsychological tasks were used, and inhibitory impairments and reaction time variability were only found in preterm and term children with ADHD.^16^ It is crucial to note, however, that this study had a limited sample of 21 preterm participants.

Based on the literature reviewed above, we aimed to assess whether ADHD imposes additional inhibitory control deficits on VP/VLBW preschool children. Since ADHD and prematurity are both associated with inhibitory control deficits, we hypothesized that premature infants with ADHD would present greater impairment in this function when compared to those without the disorder.

## Method

### Participants

**Figure 1:**
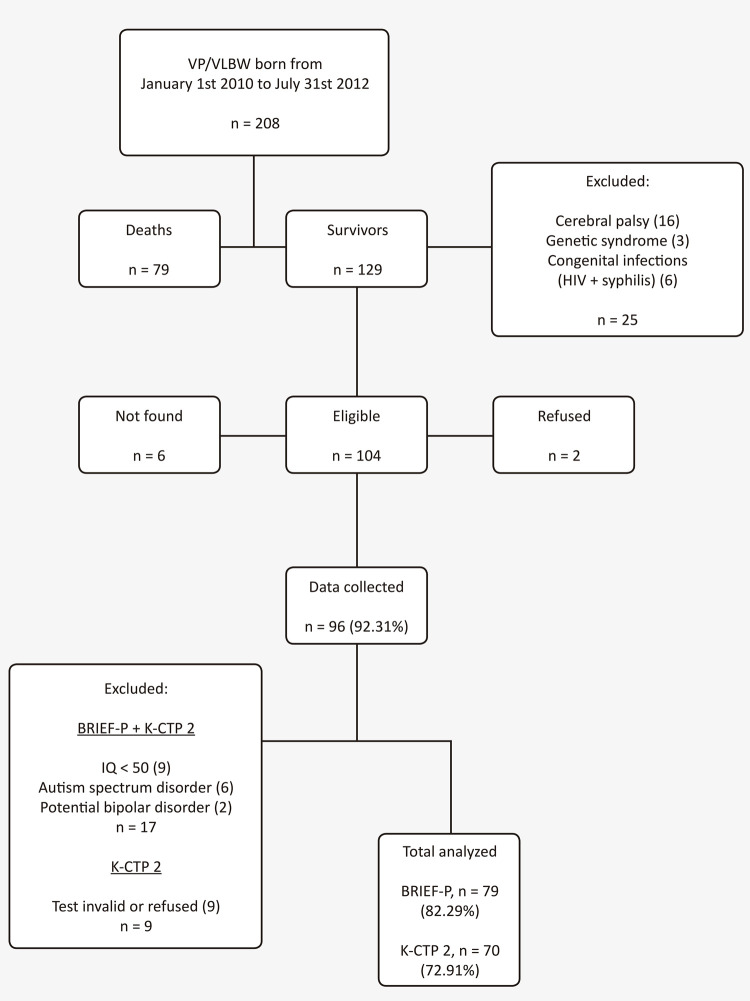
Sample selection flow diagram. BRIEF-P = Behavior Rating Inventory of Executive Function – Preschool Version; IQ = intelligence quotient; K-CPT 2 = Conners’ Kiddie Continuous Performance Test; VP/VLBW = very preterm and/or very low birth weight.

This is a case control study in which preschool children born very prematurely (less than 32 weeks) and/or with very low birth weight (under 1500g) diagnosed with ADHD were compared to those without the disorder. The sample included children between 4 and 7 years of age at the time of assessment who had previously been hospitalized at the neonatology unit of our university hospital in Porto Alegre (Hospital de Clínicas de Porto Alegre [HCPA]), in the state of Rio Grande do Sul, Brazil). The survivors among those born from January 1st, 2010 to July 31st, 2012 were eligible for the study (n = 129). Children diagnosed with either a genetic syndrome, cerebral palsy, or congenital infections (HIV or syphilis) were excluded from the sample (n = 25; see Figure 1 ). Two families of children in this sample (n = 104) refused to participate and 6 could not be located, so data were collected from 96 families. For the purposes of this study, children with an intelligence quotient (IQ) lower than 50 and those diagnosed with bipolar disorder or autism spectrum disorder were excluded. Thus, our final sample comprised 79 children for whom parents provided valid responses to the Behavior Rating Inventory of Executive Function – Preschool Version (BRIEF-P) and 70 preschoolers with valid Conners’ Kiddie Continuous Performance Test (K-CPT 2) scores (see Figure 1 ).

### Data collection and diagnostic procedures

Eligible participants were selected from the neonatology service’s records. They were contacted by phone calls, e-mail, mail, social media, or home visits. Data were collected at the hospital or at the family residence and took an average of two hours with the parents and 30 minutes with the children. The diagnostic process relied on use of a semi-structured interview, the Schedule for Affective Disorders and Schizophrenia for School Aged Children – Present and Lifetime Version (K-SADS-PL),^17^ administered by a trained child psychiatrist and answered by the child’s parents.

Participants provided verbal agreement to participation in the study and parents provided written informed consent. This study was approved by the ethics committee at the HCPA (protocol number 15-0384).

### Neuropsychological assessment

Trained psychologists assessed children in a single session. IQ was estimated using a short version^18^ of the Wechsler Preschool and Primary Scale of Intelligence (WPPSI)^19^ composed of four subtests: block design, comprehension, picture completion, and arithmetic. Inhibitory control was measured using a computerized task (K-CPT 2), performed by the children, and a behavioral inventory (BRIEF-P), responded by parents or legal guardians. These instruments are described below:

- Conners’ Kiddie Continuous Performance Test (K-CPT 2)^20^ is a computerized task designed to assess inhibitory control, sustained attention and visual-motor speed. The participant is asked to respond (by pressing the space bar) to all targets presented on a computer screen, but refrain from responding to non-targets.
- The Behavior Rating Inventory of Executive Function – Preschool Version (BRIEF-P) evaluates daily behaviors associated with specific domains of executive functioning, based on parents’ reports. Three indexes based on theoretical and empirical factor analysis findings are provided^21^ : an inhibitory self-control index (ISCI), a flexibility index (FI), and an emergent metacognition index (EMI). A translated Brazilian Portuguese version was adapted and provided by the publisher, Psychological Assessment Resources (PAR Inc.).

### Statistical analysis

Descriptive analyses were conducted to characterize the sample. Potential confounders investigated were age, IQ, gender, gestational age, socioeconomic status, and comorbidities (anxiety disorders and oppositional defiant disorder [ODD]), using the chi-square test for categorical variables and the *t* test for continuous variables. Variables with p ≤ 0.10 were included in the model (IQ and ODD). Since neuropsychological test scores are influenced by developmental aspects, age was also included in the models because scores were analyzed uncorrected for age. K-CPT 2 and BRIEF-P scores were analyzed using two independent analyses of covariance (ANCOVAs), comparing ADHD against non-ADHD groups. The analyses were performed using version 18.0 of the Statistical Package for the Social Sciences for Windows (SPSS Inc., Chicago, IL, USA) and p < 0.05 was considered significant. Effect size was calculated using partial eta squared and its magnitude defined according to the following interpretation: eta squared < 0.06 = small; 0.06-0.14 = medium, > 0.14 = large effect size.^22^ The frequency of cases with worse performance (> 1 standard deviation from normative data) was also calculated, and between-group (ADHD vs. non-ADHD) differences were investigated using chi-square tests. Normative scores for both tests were obtained respectively from a Brazilian sample^23^ and the American sample used for the original BRIEF-P manual.^21^

## Results

Data from 79 participants were analyzed for BRIEF-P scores. Nine participants refused to finish the K-CPT 2, or their assessments were considered invalid, resulting in a total sample of 70 children with K-CPT 2 data. Demographic and clinical data can be found in Table 1 . ADHD and non-ADHD groups did not differ in terms of age, gender, IQ, gestational weeks, or socioeconomic status. The frequency of anxiety disorders was also similar between groups, but the ADHD group had significantly higher rates of ODD, which is in line with previous literature.^24^

**Table 1:** Sample description

	BRIEF-P (n = 79)			K-CPT 2 (n = 70)		
	
	ADHD (n = 24)	Non-ADHD (n = 55)	p-value	ADHD (n = 21)	Non-ADHD (n = 49)	p-value
Age (years), mean (SD)	5.58 (0.92)	5.46 (0.72)	0.257	5.81 (0.90)	5.48 (0.73)	0.114
Intelligence quotient, mean (SD)	69.88 (15.87)	76.64 (17.20)	0.104	70.52 (16,58)	78.04 (17.44)	0.098
Gestational age (weeks), mean (SD)	30 (2.53)	30.27 (2.49)	0.678	30.11 (2.68)	30.37 (2.42)	0.710
SES, mean (SD)	24.08 (7.76)	26.40 (7.57)	0.218	23.90 (7.58)	26.57 (7.53)	0.180
Gender (male), n (%)	12 (50)	26 (47.27)	0.823	11 (52.38)	22 (44.90)	0.565
Oppositional defiant disorder, n (%)	11 (45.83)	6 (10.90)	0.001	6 (28.57)	9 (18.37)	0.004
Any anxiety disorder, n (%)	7 (29.17)	13 (23.64)	0.603	5 (23.81)	13 (26.53)	0.811

ADHD = attention-deficit/hyperactivity disorder; SD = standard deviation; SES = socioeconomic status (higher is better).

Findings emerging from ANCOVAs did not reveal significant differences between ADHD and non-ADHD groups, either for any of the BRIEF-P scores or for K-CPT 2 variables (p = 0.062 to p = 0.903). All effect sizes were small ( Table 2 ). Correlations between K-CPT 2 and BRIEF-P scores were all non-significant and ranged from -0.197 to 0.121.

**Table 2:** ADHD vs. non-ADHD performance in measures of executive function

	ADHD Mean (SE)	Non-ADHD Mean (SE)	p-value	ES
BRIEF-P*	(n = 24)	(n = 55)		
Inhibitory self-control	40.13 (1.87)	35.46 (1.62)	0.062	0.046
Flexibility	26.6 (1.38)	26.32 (1.2)	0.880	0.001
Emergent metacognition	40.60 (2.01)	39.72 (1.74)	0.740	0.002
K-CPT 2*	(n = 21)	(n = 49)		
Omissions	40.58 (7.21)	36.29 (5.78)	0.637	0.004
Commissions	104.69 (10.86)	103.03 (8.70)	0.903	0.001
Perseverations	7.18 (2.43)	8.89 (1.95)	0.576	0.005
HRT	726.37 (38.11)	693.73 (30.56)	0.496	0.007
HRT SD	374.32 (27.58)	361.91 (22.11)	0.720	0.002
Variability	112.66 (12.48)	127.74 (9.99)	0.336	0.017
HRT block change	-0.960 (9.39)	12.48 (7.77)	0.264	0.020
HRT ISI	112.30 (21.68)	77.78 (17.39)	0.208	0.025

ADHD = attention deficit/hyperactivity disorder; BRIEF-P = Behavior Rating Inventory of Executive Function – Preschool Version; ES = effect size; K-CPT 2 = Conners’ Kiddie Continuous Performance Test; HRT = hit reaction time; HRT ISI = hit reaction time interstimulus-interval; HRT SD = hit reaction time standard deviation; SE = standard error.

* Potential confounders: age, estimated intelligence quotient, oppositional defiant disorder; all measures are raw scores.

The frequency of cases with worse performance (> 1 standard deviation [SD] from the normative mean) on both instruments was also analyzed. Regarding BRIEF-P scores, 12 children (15.2%) satisfied this condition for the ISCI. For the FI and the EMI, 6 (7.6%) and 17 (21.5%) children respectively were included in the groups with the worst performance. No association was found between ADHD and worse performance in BRIEF-P indexes (see Table 3 ).

**Table 3:** Executive function deficits in ADHD vs. non-ADHD children

	ADHD (n = 24)		non-ADHD (n = 55)		
	
	> 1 SD	≤ 1 SD	> 1 SD	≤ 1 SD	p-value
Inhibitory self-control	6 (25)	18 (75)	6 (10.91)	49 (89.09)	0.109
Flexibility	1 (4.17 )	23 (95.83)	5 (9.09)	50 (90.91)	0.447
Emergent metacognition	7 (29.17)	17 (70.83)	10 (18.18)	45 (81.81)	0.275
	
	**n = 21**		**n = 49**		
	
Omissions	12 (57.14)	9 (42.86)	26 (53.06)	23 (46.93)	0.753
Commissions	8 (38.1)	13 (61.90)	23 (46.94)	26 (53.06)	0.495
Perseverations	5 (23.81)	16 (76.19)	10 (20.41)	39 (79.60)	0.751
HRT	7 (33.33)	14 (66.67)	13 (26.53)	36 (73.47)	0.564
HRT SD	20 (95.24)	1 (4.76)	49 (100)	0 (0)	0.124
Variability	17 (80.95)	4 (19.05)	43 (87.76)	6 (12.24)	0.456
HRT block change	9 (42.86)	12 (57.14)	33 (67.35)	16 (32.65)	0.055
HRT ISI	16 (76.2)	5 (23.81)	39 (79.6)	10 (20.41)	0.751

Data presented as n (%).

ADHD = attention deficit/hyperactivity disorder;; HRT = hit reaction time; HRT block change = hit reaction time block change; HRT ISI = hit reaction time interstimulus-interval; HRT SD = hit reaction time standard deviation; SD = standard deviation.

Regarding worse K-CPT 2 performance (defined as > 1 SD from the normative mean), 69 children (98.6%) fulfilled this condition for the hit reaction time standard deviation (HRT SD) index, 60 (85.7%) for variability, 42 (60%) for hit reaction time block change (HRT block change), and 55 (78.6%) for hit reaction time interstimulus-interval (HRT ISI). The numbers of children classified with worse performance according to the results for the indexes were as follows: omissions 38 (54.3%), commissions 31 (44.3%), perseverations 15 (21.4%), and HRT 20 (28.6%). We also did not find an association between ADHD and worse performance in the K-CPT 2 scores (see Table 3 ).

## Discussion

Consistent findings of behavioral and/or cognitive profiles in preterm born children have provided evidence of a “preterm phenotype”. Psychiatric disorders, especially ADHD, anxiety, and autism spectrum disorder, are the most prevalent among subjects born preterm. This preterm behavioral phenotype is usually found in association with cognitive impairment.^25^ Investigations with adults born prematurely at VLBW support these findings and show that preterm birth has long-term consequences,^26^ including neurocognitive deficits.^27^

Specifically with relation to inhibitory control, current evidence shows that a deficit is present both in ADHD,^8 , 10 , 13 , 14^ and in children born very prematurely.^3 , 28^ However, to our knowledge, only one study has compared inhibitory control in VP/VLBW children with and without ADHD. Contrary to our original hypothesis, our results revealed no significant differences in inhibitory control between VP/VLBW children with and without ADHD.

In general, weak correlations are found between tests of executive function performance and behavior scales in the literature.^29^ The type of measurement selected (behavioral reports or laboratory measures) can also influence the results, either by their intercorrelations or by their associations with ADHD.^30^ This is why we used both a performance measure (K-CPT 2) and a parent-rated behavior scale (BRIEF-P). We found consistent results across instruments, indicating no differences between ADHD and non-ADHD groups. Also, as described in the literature, correlations between the two types of measures were low.

Given this finding, we also analyzed whether the groups’ results were 1 SD or more from the normative mean, indicating a deficit in comparison to the normative samples for K-CPT2 and BRIEF-P. We chose a lenient threshold, of one standard deviation or 15.9% from the normal distribution mean, to identify cases that showed even a mild difficulty. The BRIEF-P results indicated that the ADHD group had a slightly higher frequency than that predicted by a normal distribution in the inhibitory self-control and emergent metacognition scales. This was only observed for emergent metacognition in the non-ADHD group. Nevertheless, the differences were not significant, and at least 70.83% of the children had performance considered to be in the normal range in both groups. Although previous studies show that EF deficits are present in ADHD and VP/VLBW patients, rating scales focus on a global and less specific observation of executive functioning in the everyday context. They also depend on parent ratings, which might be biased by different development expectations.^31^ This could have influenced our results since a global developmental delay characterizes our sample.

Overall, K-CPT 2 scores indicated a higher number of participants in the clinical-suggestive range, with no significant differences between groups. Omissions, commissions, HRT, and HRT block change results indicated that 26 to 67% of the children had performance more than one standard deviation from the normative mean, independent of group status. These measures are related to inattention, impulsivity, speed of processing, and vigilance, respectively. With regard specifically to commissions, which is a measure related to impulsivity and inhibitory control, 38% of the ADHD group and 46% of the non-ADHD group fell within the clinical range, as suggested by the literature.^10 , 32^

Regarding reaction time variability (RTV), 76.2% to 100% of the sample had impaired performance and once again no differences were found between groups. This variability is associated with most childhood psychiatric disorders, traumatic brain injury, dementia, and aging populations.^32 , 33^ Few studies have investigated this issue in premature children. Adolescents born prematurely show an impairment in reaction time variability when compared to controls. Regardless, this impairment is milder than the impairment observed in term-born ADHD adolescents.^15^ However, this result was not observed in the same sample when a different cognitive test was analyzed.^34^ Although this construct still remains understudied in prematurity, it is well established that a higher than usual RTV is present in ADHD and is also a marker of general psychopathology.^33 - 35^ The pathophysiology of RTV is usually associated with abnormal frontal lobe volume and/or activation.^33^ Cortical maturation of the frontal lobe is delayed in ADHD children,^36^ and a meta-analysis showed an overall reduction in brain volume, including white and gray matter in VP/VLBW school-aged children.^37^ Specifically in preschool children, a delay in normal cortical and surface development is associated with prematurity.^38^

Another critical aspect to discuss is that our sample was characterized by a below-average mean IQ, irrespective of group. However, this is not an unexpected finding since low birth weight is also associated with below-average IQ. The high degree of nervous system immaturity and the greater susceptibility to neonatal complications may lead to cognitive impairment. The cerebral networks of children with low birth weight are less connected brain networks, with lower brain volumes and lower cortical surface area, which might result in impaired cognitive functions.^2 , 39^ Moreover, results of a meta-analysis also showed that ADHD is associated with lower overall cognitive ability when compared to healthy controls.^40^

The nature of the relationship between intelligence and executive functions is still controversial.^41^ Although many variables are included in the broader concept of executive functions, they are separable to a certain degree and differently correlated to each other as well as to the many areas of the brain.^42^ Among all other executive functions, inhibition has been found to have a low correlation to IQ.^43^ The same is valid for RTV: although higher inconsistency is related to lower IQ, the variance explained by IQ is usually small.^44^ A recent meta-analysis showed evidence for a low to moderate correlation with matrix intelligence tests - which are less affected by cultural aspects.^45^ Considering the above, the participants’ low IQ may only partially explain the deficits in inhibitory control and RTVs found in our sample.

Our findings should be understood in the context of some limitations. Although the low average IQ observed in our sample is a common finding in VP/VLBW children and it may not fully explain our results, we cannot discard it as a limitation. Further studies investigating the performance of average-IQ preterm/low-birth preschoolers might improve our understanding of the impact of ADHD on executive functions in this population. Additionally, our moderate sample size in both groups might have decreased the power for detection of between-group differences. However, it is essential to note that all between-group ES were small for both BRIEF-P and K-CPT 2 scores.

Furthermore, the absence of a full-term control sample imposes limitations on our analyses, making us rely on normative data for comparisons. Still, regarding K-CPT 2 and BRIEF-P, validity studies for the Brazilian population were not available. Finally, another aspect we did not adequately control was use of medications. Although none of the participants were using ADHD medication, we do not have information accurately recorded in our dataset about other clinical medications that might interfere with inhibitory control.

## Conclusions

This study provides evidence of performance deficits on inhibitory control tests in preschool children born VP and/or with VLBW. Presence of ADHD did not impose any additional burden on the children’s test performance. Parents’ reports seem to be less sensitive for capturing executive deficits in this population. Impairment in RTV, a more basic cognitive process, was found in most participants, independent of group status. Our findings suggest either that assessment of EFs using neuropsychological tasks and parent reports may not be sufficiently sensitive to differentiate between ADHD and non-ADHD in VP/VLBW preschool children, or that these children already have a level of impairment in executive functions that does not leave much room for additional impairment. More large-scale studies investigating the nature and long-term effects of inhibitory control deficits in these vulnerable populations are needed.
